# Insights into the *Geobacillus stearothermophilus* species based on phylogenomic principles

**DOI:** 10.1186/s12866-017-1047-x

**Published:** 2017-06-26

**Authors:** S. A. Burgess, S. H. Flint, D. Lindsay, M. P. Cox, P. J. Biggs

**Affiliations:** 1 School of Food and Nutrition, Massey University, Palmerston North, New Zealand; 2 Fonterra Research Institute, Palmerston North, New Zealand; 3 Statistics and Bioinformatics Group, Institute of Fundamental Sciences, Massey University, Palmerston North, New Zealand; 4 Infectious Disease Research Centre, Institute of Veterinary, Animal and Biomedical Sciences, Massey University, Palmerston North, New Zealand

**Keywords:** *Geobacillus*, Thermophile, Dairy, Comparative genomics

## Abstract

**Background:**

The genus *Geobacillus* comprises bacteria that are Gram positive, thermophilic spore-formers, which are found in a variety of environments from hot-springs, cool soils, to food manufacturing plants, including dairy manufacturing plants. Despite considerable interest in the use of *Geobacillus* spp. for biotechnological applications, the taxonomy of this genus is unclear, in part because of differences in DNA-DNA hybridization (DDH) similarity values between studies. In addition, it is also difficult to use phenotypic characteristics to define a bacterial species. For example, *G. stearothermophilus* was traditionally defined as a species that does not utilise lactose, but the ability of dairy strains of *G. stearothermophilus* to use lactose has now been well established.

**Results:**

This study compared the genome sequences of 63 *Geobacillus* isolates and showed that based on two different genomic approaches (core genome comparisons and average nucleotide identity) the *Geobacillus* genus could be divided into sixteen taxa for those *Geobacillus* strains that have genome sequences available thus far. In addition, using *Geobacillus stearothermophilus* as an example, we show that inclusion of the accessory genome, as well as phenotypic characteristics, is not suitable for defining this species. For example, this is the first study to provide evidence of dairy adaptation in *G. stearothermophilus* - a phenotypic feature not typically considered standard in this species - by identifying the presence of a putative *lac* operon in four dairy strains.

**Conclusions:**

The traditional polyphasic approach of combining both genotypic and phenotypic characteristics to define a bacterial species could not be used for *G. stearothermophilus* where many phenotypic characteristics vary within this taxon. Further evidence of this discordant use of phenotypic traits was provided by analysis of the accessory genome, where the dairy strains contained a putative *lac* operon. Based on the findings from this study, we recommend that novel bacterial species should be defined using a core genome approach.

**Electronic supplementary material:**

The online version of this article (doi:10.1186/s12866-017-1047-x) contains supplementary material, which is available to authorized users.

## Background

The *Geobacillus* genus contains Gram-positive, rod-shaped, spore-forming bacteria that have an optimum growth temperature of 55–65 °C [1]. Members of the *Geobacillus* genus were originally classified in Group 5 of the *Bacillus* genus [2]. In 2001, based on a combination of 16S ribosomal RNA (rRNA) sequence analysis, fatty acid composition and DNA-DNA hybridization (DDH), some members of Group 5 were reclassified into the new genus *Geobacillus*, with the word *Geobacillus* meaning “soil or earth small rod” [1]. Recently it was proposed that the *Geobacillus* genus be separated into two genera based on a comparative genomics analysis, which we explore further here [3]. There is extensive interest in the *Geobacillus* genus for biotechnological purposes such as for bioremediation, the production of thermostable enzymes, and biofuels [4–7]. In addition, *Geobacillus* spp. are common spoilage organisms in food manufacturing plants and products [8–14]. *Geobacillus* spp. have been isolated from temperate as well as hot environments including hot springs, oilfields, deep sea sediments, sugar refineries, canned foods, dehydrated vegetables and dairy factories. The species *G. stearothermophilus* was first described in 1920 and was isolated from canned cream-style corn. *G. stearothermophilus* is a common contaminant of dairy products, particularly milk powder and has also been isolated from dried soups and vegetables. Until the 1980s *G. stearothermophilus* was regarded as the only known obligate thermophile of the *Bacillus* genus [15, 16].

According to the LPSN bacterio.net [17], as of April 2017, there were sixteen *Geobacillus* species (*G. caldoxylosilyticus, G. galactosidasius, G. icigianus, G. jurassicus, G. kaustophilus, G. lituanicus, G. stearothermophilus, G. subterraneus, G. thermantarcticus, G. thermocatenulatus, G. thermodenitrificans, G. thermoglucosidasius, G. thermoleovorans, G. toebii, G. uzenensis* and *G. vulcani*) described with validly published names [1, 18–27]. However, the classification of many of these species remains uncertain. To date over 60 *Geobacillus* genomes have been sequenced, mainly to identify genes that could be used in different biotechnological applications [3]. Of these, there are eleven species with genome sequences of the type strain (*G. caldoxylosilyticus* NBRC 10776, *G. icigianus* DSM 28325, *G. jurassicus* DSM 15726, *G. kaustophilus* NBRC 102445, *G. stearothermophilus* ATCC 12980, *G. subterraneus* DSM 13552, *G. thermoantarcticus* M1, *G. thermodenitrificans* DSM 465 *G. thermoglucosidasisus* NBRC 107763, *G. thermoleovorans* DSM 5366, and *G. toebii* DSM 14590) [3, 28, 29]. Recent studies have shown that it is possible for a comparative genomics approach to resolve the taxonomy of this important genus [3, 30]. However, the question still remains as to the most appropriate genomics tool for the classification of new species.

Despite the advances of the post-genomics age, there is still no consensus as to what characterizes a bacterial species [31, 32]. However, in describing a new bacterial species, the two methods on which the most emphasis has been placed are 16S rRNA gene sequence analysis and DDH, alongside various phenotypic methods [33]. However, in some cases, including the *Geobacillus* genus, the sequence similarity of the 16S rRNA is >97% between species despite being distinct when the overall genome DNA similarity is analyzed using DDH [34–38]. Therefore the identification of new *Geobacillus* species generally relies on other approaches, such as DDH.

In general, DDH is also fraught with challenges as a method for the differentiation of bacterial species because it is laborious and there is a lack of reproducibility, reciprocation, and calibration of the method with a reference strain of a known DDH value [39–41]. In the case of new *Geobacillus* species, DDH values between studies show large variations [21, 27], which has led to the reclassification of some species of *Geobacillus*. Dinsdale et al. [21] showed that some of the previously published species were in fact synonymous with current species and should no longer be considered valid. For example, the described species *G. kaustophilus* [1, 38, 42], *G. lituanicus* [23] and *G. vulcani* [43] were shown to be synonymous with *G. thermoleovorans*. In addition, the described species *G. gargensis* [27] was synonymous with *G. thermocatenulatus*. Most of the disagreement in assigning new species to the *Geobacillus* genus comes from the DDH values used to distinguish strains being very different between studies. More recently it was proposed that the strains of *G. kaustophilus* and *G. thermoleovorans* should both be designated to the *G. thermoleovorans* species [3].

Other housekeeping genes, such as *recN*, *recA*, *rpoB*, *gyrB*, *parE* and *spo0A*, have been evaluated as alternatives to the 16S rRNA gene for identifying *Geobacillus* species, all with limited success [37, 44–46]. Of the genes analyzed, *recN* appears to be the most reliable, with a higher taxonomic resolution compared with 16S rDNA [46]. However, the taxonomic resolution between some species of *Geobacillus* is still poor (for example, between *G. subterraneus* and *G. uzenensis*). This is not surprising given that house-keeping genes are well conserved between closely related species, and relying on one or a few genes does not depict the real diversity of the entire genome.

In the era of next generation sequencing it is likely that DDH will become outdated. This is already apparent with the proposal to use comparative genomics approaches to demarcate new species with genomic DNA as the type material archived alongside live cultures [47, 48]. There are a number of different ways in which whole genome sequence data can be used in taxonomy; for example, average nucleotide identity (ANI), tetranucleotide frequency, core genome analysis, pan genome analysis, and multilocus sequence typing (MLST) [49]. There appear to be two schools of thought on how a genomics based method should be incorporated into prokaryotic taxonomy. Firstly, there is a traditional polyphasic approach that incorporates both genomic as well as phenotypic characteristics [50]. In this case, the most likely substitute for DDH is ANI [33, 47]. It has been shown that an ANI value of <95–96% generally corresponds well with the thresholds of <70% for DDH and <97–98% for 16S rRNA gene identity for defining new species [40, 51]. Secondly, there is a reliance on a genomic approach only, simply using a core genome analysis or a combination of core genome and ANI [52, 53].

Until recently, none of the broader taxonomic studies on the *Geobacillus* genus have included *G. stearothermophilus* strains of dairy origin as part of their comparison. Traditionally both a genotypic and phenotypic analysis is carried out to identify a new species. However, the relationship between phenotype and genotype is not always straightforward. This is particularly well exemplified with dairy strains of *G. stearothermophilus*, which show unique physiological characteristics such as their metabolism (e.g. the ability to utilize lactose), and the fatty acid profile from the type strain *G. stearothermophilus* ATCC 12980 [54]. Differences in phenotypic traits may therefore result from niche adaptation, possibly mediated by differential gene expression, without major changes to the genome as a whole.

The aims of this study were two-fold: firstly, to establish whether the species boundaries of the *G. stearothermophilus* taxon could exclusively be determined by whole-genome sequence analyses, and secondly to determine whether the genomes of the dairy strains of *G. stearothermophilus* provide evidence of niche adaptation in ways that deviate from the standard phenotypic spectrum of the species. To pursue these goals, we compared the genome sequences of 63 *Geobacillus* strains, including twelve *G. stearothermophilus* strains, of which four were isolated from a dairy manufacturing environment.

## Results

To gain an understanding of how the *G. stearothermophilus* strains isolated from dairy manufacture are related to other *Geobacillus* species, two different phylogenomic approaches were taken: ANI and a comparison of the core genomes. In addition, these methods were evaluated for their ability to replace the traditional methods of DDH, 16S rRNA sequence analysis and phenotypic characteristics to define a bacterial taxon, using *G. stearothermophilus* as an exemplar. The genomes of 63 *Geobacillus* strains (including ten type strains) were compared, of which eight strains were originally isolated from a dairy manufacturing environment or food product. Four of the eight strains were *G. stearothermophilus*, three of which were isolated from a New Zealand milk powder manufacturing and the fourth was isolated in the Netherlands from buttermilk powder [55, 56]. Within the *Geobacillus* genus the *G. stearothermophilus* type strain ATCC1290 had the smallest genome (2.63 Mb) compared with the dairy strain *G. caldoxylosilyticus* B4119 which has the largest genome size (3.95 Mb) within the *Geobacillus* genus (Additional file 1: Table S1). The genome sizes of the *G. stearothermophilus* dairy strains ranged from 2.77 to 3.02 Mb.

### Phylogenetic relationships within the *Geobacillus* genus based on core genome comparisons

**Fig. 1:**
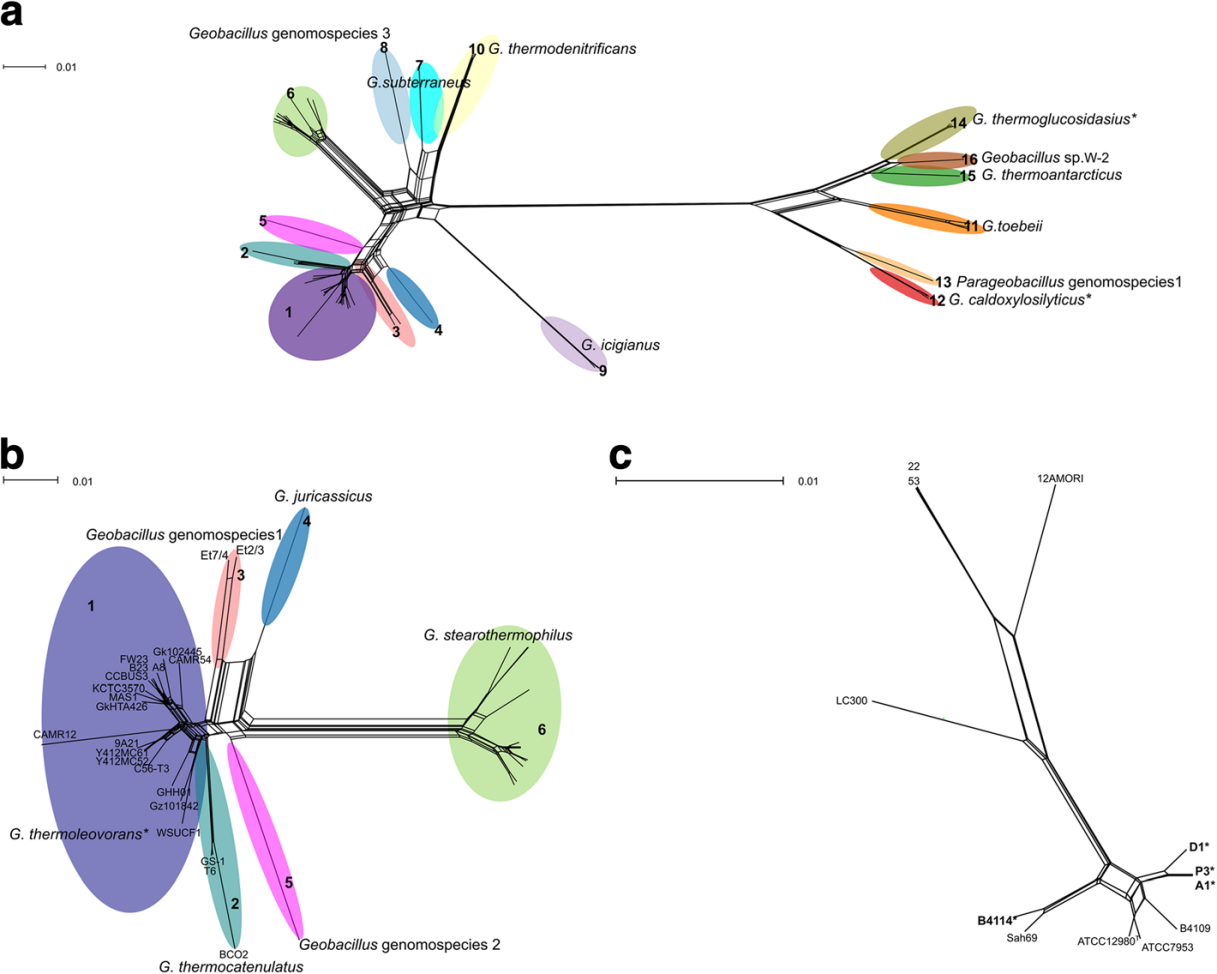
Core genome sequence comparisons. The phylogenetic networks were generated using the Neighbor-Net algorithm in SplitsTree (v. 4.13.1). The orthologous groups were defined using the program OrthoMCL (v. 2.0.9) and the analyses were based on those genes that have orthologous gene members with a length range less than or equal to 20%. Those groups marked with an asterisk contain a strain(s) that originate from a dairy environment. **a**. Includes all of the *Geobacillus* genomes where *G. thermoleovorans* group 1 refers to strains B23, CCB_US3_UF5, CAMR5420, FW23, MAS1, NBRC 102445, A8, Y412MC52, Y412MC61, 9A21, C56-T3, GHH01, WSUCF1 and HTA426, KCTC 3570, and *G. zalihae* NBRC 101842; Group 2 refers to “*G. thermocatenocatulatus”* strains GS-1, T6, and BC02; Group 3 refers to *Geobacillus* genomospecies 1 strains Et7/4 and Et2/3; Group 4 refers to *G. juracassicus*; Group 5 refers to *Geobacillus* genomospecies 2 strain PSS1; *G. stearothermophilus* Group 6 includes strains ATCC 12980, ATCC 7953, A1, P3, D1, B4114, 22, 53, Sah69, 12AMORI, LC300 and B4109; *G. subterraneus* Group 7 includes strains KCTC 3922 and K; Group 8 refers to *Geobacillus* genomospecies 3 strain JF8; *G. icigianus* Group 9 refers to strains PSS2 and G1w1T; *G. toebii* Group 10 includes strains NBRC 107807, WCH70 and B4110; *G thermodenitrificans* Group 11 includes strains DSM 465, NG80–2, PA3 and G11MC16; *G*. *caldoxylosilyticus* Group 12 includes strains CIC9, B4119 and NBRC 10776; Group 13 refers to *Parageobacillus* genomospecies 1 strain NUB3621; *G. thermoglucosidasius* Group 14 includes strains NBRC 10776, C56YS93, TNO-09 and Y4.1MC1; *G. thermoantarcticus* Group 15 includes strain M1; Group 16 includes strain W-2. **b**. Includes Groups 1–6. **c**. Includes *G*. *stearothermophilus* Group 6 only

A core genome analysis was used to determine phylogenetic relationships within the *Geobacillus* genus and to establish the species boundary of the *G. stearothermophilus* taxon. The core genome was defined using the program OrthoMCL, in which each orthologous group contained only one gene from each genome. In addition, to be included in the core genome, the length range (between the smallest and the largest) of the amino acid sequences within each cluster was not allowed to vary by more than 20%. A phylogenetic network was then generated using the concatenated sequence of those orthologous genes (Fig. 1). Core genome comparisons separated the *Geobacillus* genus (Subset A, Table 1) into sixteen main groups and several sub-groups. Genomes of strains isolated from a dairy environment, indicated by asterisks, included strains of *G. thermoglucosidasius*, *G. caldoxylosilyticus*, *G. kaustophilus* and the focus of this study *G. stearothermophilus*.

**Table 1:** Number of genes and amino acids used in the OrthoMCL clustering

Subset^a^	Members of group	Same length and same sequence		Same length and different sequence		Cluster length^b^		Total number of core genes
		Genes	Amino acids	Genes	Amino acids	Genes	Amino acids	
A	All *Geobacillus*	1	116	58	7678	390^c^	81,812	391
B	Groups 1 – 6^d^	7	865	131	19,180	478^c^	99,197	485
C	*G. stearothermophilus* group	138	19,490	992	277,724	1524^c^	446,984	1662

^a^Refer also to Fig. 1

^b^To be in an orthologous cluster, genes had to have a length range of 20% across all cluster members, and only one member per strain

^c^This number of genes was used in the Neighbor-Net analysis

^d^Refer to Fig. 1 for strains included in each group

^e^Includes strains ATCC 12980, ATCC 7953, LC300, 12AMORI, 22, 53, Sah69, A1, P3, D1, B4109, B4114

To analyze the relationship of the *G. stearothermophilus* taxon more closely, comparison of the core genome was carried out on two smaller groups of *Geobacillus* taxa (Subset B and C, Table 1, Fig. 1b and c). There is a clear delineation between the *G. stearothermophilus* cluster and other closely related *Geobacillus* taxa (Groups 1–5, Fig. 1b). Within the *G. stearothermophilus* taxon three of the dairy strains (all from the same manufacturing plant) clustered together, showing no sequence diversity between strains A1 and P3 (Fig. 1c).

### Defining taxa in *Geobacillus* on the basis of ANI calculations

**Fig. 2:**
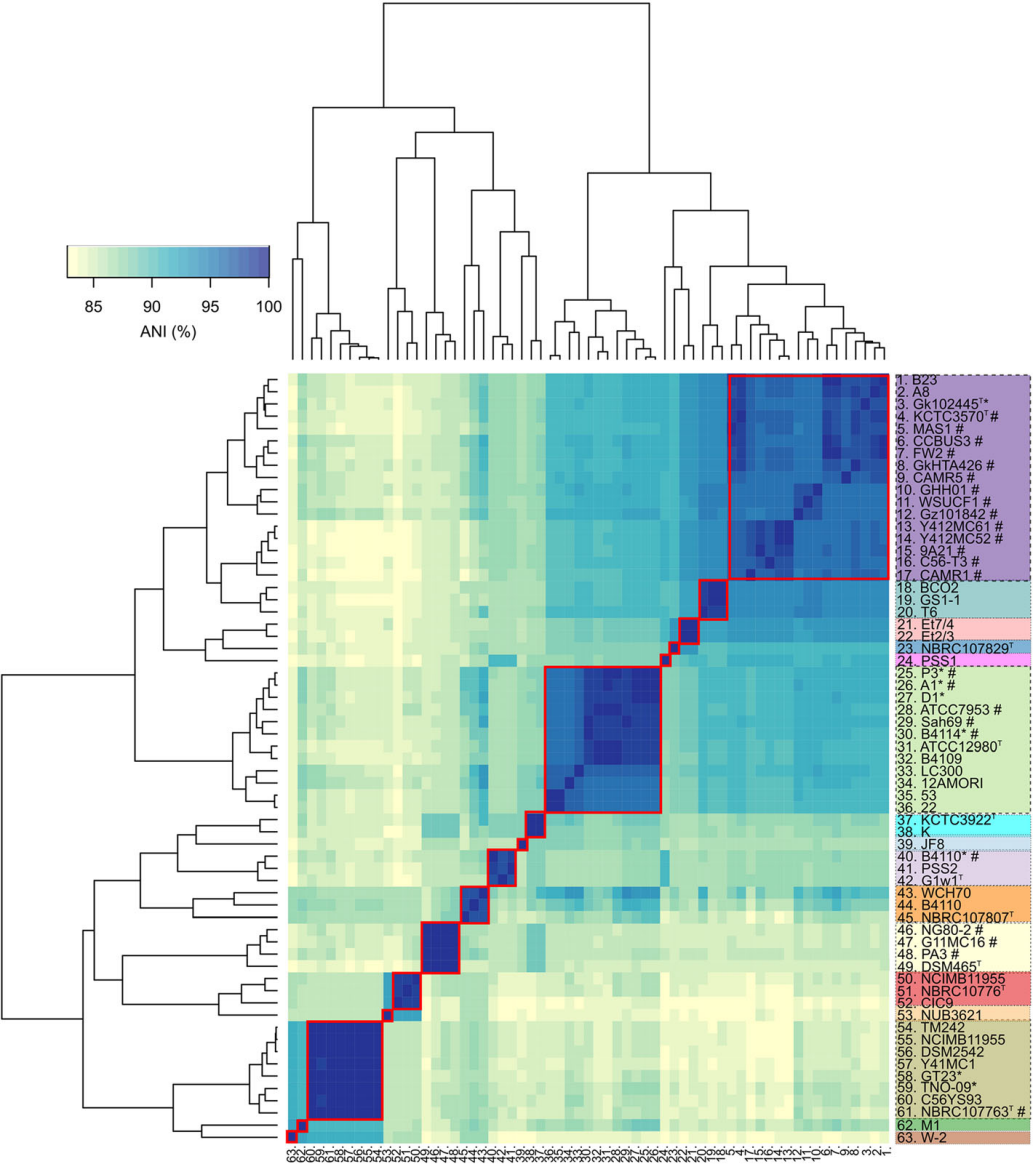
Heat map comparison of the ANIm values. Those strains marked with an asterisk were isolated from a dairy environment and those strains marked with a hash were placed in a different order for the reciprocal pairwise comparison by the dendrogram option using the heatmap.2 function in R. Those ANI values greater than 95%, grouping the strains within the same species, were enclosed by a *red box*

As stated above, the most feasible substitute for DDH is ANI [33, 47]. To examine the use of ANI for demarcating species of the *Geobacillus* genus, ANIm frequencies were calculated for all of the sequenced genomes of the *Geobacillus* genus (Additional file 1: Table S2) and visualized using a heat-map (Fig. 2). Two ANI values were calculated for each pair of genomes with one being the subject and the other the query, and vice versa. The heat-map was non-symmetrical as a result of greater differences between the ANIm value and its reciprocal value for some pairs of genomes. When the difference between two ANIm values is greater than 0.5% around the 95% threshold it could potentially place ambiguity around the taxonomic position of a strain. However, this was not seen in this study, where the difference in two ANIm values between two members of the same taxon was always less than 0.5% (data not shown), so that there were clear demarcations between taxa (as designated by a red box in Fig. 2). The *G. stearothermophilus* strains had ANIm values >95% grouping them within the same taxon.

### Phenotypic characteristics as taxonomic determinants

To date, descriptions of novel bacterial species have included unique phenotypic characteristics. However, many descriptions are based on only a small population of strains, and in some cases, only one strain. When a larger population is examined, phenotypic characteristics can often vary between strains of the same taxon [32]. This was seen within the *G. stearothermophilus* taxon (Table 2), where the use of phenotypic characteristics was not a reliable taxonomic determinant. Several phenotypic characteristics were different between the dairy strains and that described for the *G. stearothermophilus* species, as well as differences identified between the dairy strains themselves (Table 2).

**Table 2:** Phenotypic characteristics^a^ of *G. stearothermophilus* strains

Characteristic	A1^b,g^	D1^b,g^	P3^b,g^	Dairy^c^	ATCC 7953^d^	ATCC 12980^e^	Species description^f^
Motility	−	−	+	v	n/d	n/d	+
*Acid from:*							
Adonitol	−	−	−	−	−	-*n/d	−
Amidon^g^	−	+	−	v	+*	v*	n/d
Arabinose	w	−	w	w/−	−	−	v
Cellubiose^g^	+	+	+	+	v	-*	−
Fructose	+	+	+	+	+	+	+
Galactose^g^	w	+	+	w/+	−	v	−
Gentiobiose^h^	−	−	−	v	-*	v	−
Glucose	n/d	n/d	n/d	+	+	+	+
Glycerol	n/d	n/d	n/d	-	+*	v	+
Glycogen^g,h^	−	−	−	v	+*	+	+
Inositol	−	−	−	−	−	−	−
Inulin^g^	−	−	−	−	−	v	+
Lactose^g^	+	+	+	+	−	-*	−
Maltose	n/d	n/d	n/d	+	+	+	+
Mannitol	−	−	−	−	Var	−	Var
Mannose^h^	+	+	+	v	+*	+	+
Melezitose^h^	−	+	−	v	+*	+	+
Melibiose	+	+	+	v	+*	+	+
MethylD-glucoside^g, h^	−	−	−	v	n/d	+	+
Raffinose^, h^	n/d	n/d	n/d	v	v	+	+
Rhamnose	−	−	−	−	−	−	−
Ribose	−	−	−	−	-*	−	−
Salicin^g, h^	+	+	+	v	-*	v	−
Sorbitol	−	−	−	−	−	−	−
Sucrose	+	+	+	+	+	+	+
Trehalose^h^	+	−	+	v	+*	+	+
D-Turanose^h^	−	−	−	v	+*	+	v
Xylose	−	−	−	−	−	-*	v
*Utilization of:*							
Citrate	−	−	−	−	-	n/d	−
Formate				n/d			−
Lactate				n/d			−
*Hydrolysis of*							
Casein	−	−	−	-	v	n/d	v
Esculin	+	+	+	+	v	n/d	v
Gelatin^g^	−	−	−	−	+	n/d	+
Starch^g^	−	−	−		+	n/d	+
Nitrate reduction	+	+	+	+	+	n/d	v
Phenylalanine deamination	−	−	−	−	n/d	n/d	n/d
L-Pyroglutamic acid^h^	−	+	−	v	n/d	n/d	n/d
p-Nitrophenyl-β-D-glucoside^g^	+	−	+	v	n/d	n/d	n/d

^a^Abbreviations are as follows: v, variable; w, weak reaction; n/d, not described

^b^Data from this present study and Burgess et al. [54]

^c^Data from Flint et al. [10] and Burgess et al. [54]

^d^Data from this present study (marked as *), Baldock [95], Humbert et al. [96] and Jung et al. [97]

^e^Data from this present study (marked as *), Walker and Wolf [98], Logan and Berkeley [99] and Flint et al. [10]

^f^As described by Logan et al. [70]

^g^Phenotypic characteristic that is variable between the dairy strains

^h^Phenotypic characteristic that is different between the dairy strains and that described for the *G. stearothermophilus* species

### Unique accessory genes required for adaptation to a dairy environment

**Fig. 3:**
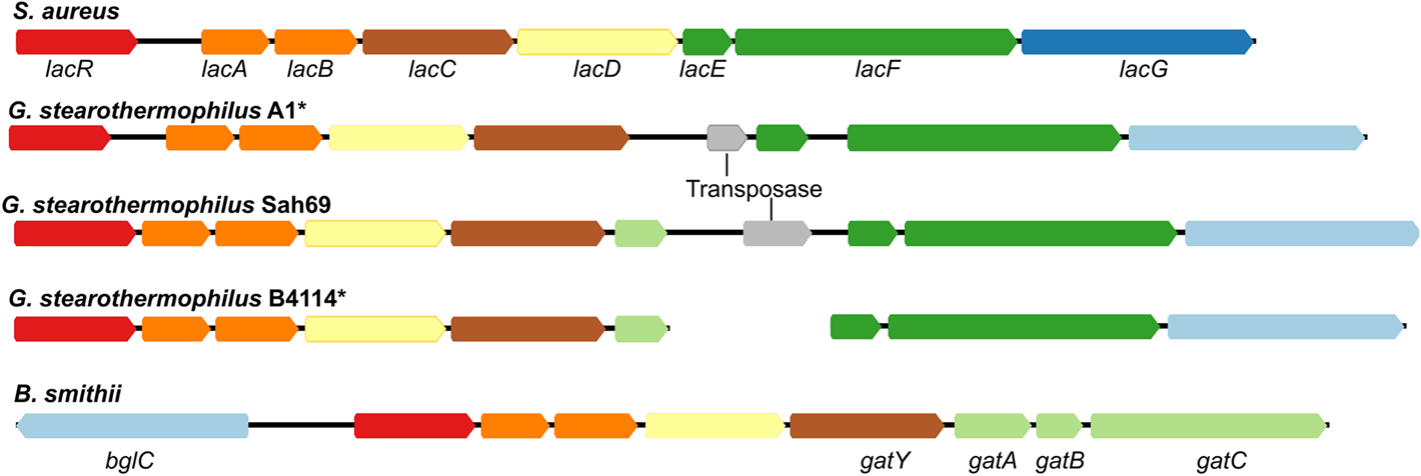
Comparison of the organisation of the *lac* genes. Annotations are based on the assigned KEGG KO for each gene. Colours represent those genes belonging that to the same KO group and/or KEGG enzyme entry. The *lac* operon in *S. aureus* and the putative *lac* operons in strains A1, Sah69, B4114 as well as *B. smithii* (which showed the highest similarity to the putative *lacA*, *lacB* and *lacC* genes from strain A1). Those strains marked with an asterisk were isolated from a dairy environment. The gene organisation of the putative *lac* operon in strains P3 and D1 was syntenic with that of A1. The *gatABC* operon encodes a galactitol transport system and *gatY* a component of the of the GatYZ tagatose aldolase as described byVan der Heiden et al. [82]. GatY and LacD both belong to the same enzyme group (EC 4.1.2.40)

Recently the genomes of four dairy strains of *G. stearothermophilus* have been sequenced [54, 55]. The accessory genomes of these four strains were analyzed to determine whether the presence or absence of genes or gene clusters could account for any of the phenotypic differences observed between the dairy strains and the type strain (ATCC 1294). A putative *lac* operon was identified in the dairy strains of *G. stearothermophilus* that was not found in any of the other *Geobacillus* genomes analysed, with the exception of *G. stearothermophilus* strain Sah69 that originates from soil. For all four dairy strains and strain Sah69, the putative *lacA*, *lacB* and *lacC* genes showed highest homology (95–99% amino acid identity) with *Bacillus smithii* and the *lacE*, *lacF* and *bglC* genes showed highest homology (70–79% amino acid identity) with *Bacillus cereus*. The gene organisation of these *lac* operons were compared with the *lac* operon of *Staphylococcus aureus*, and as seen in Fig. 3, they are missing the *lacG* gene, which encodes a galactosidase, required for splitting lactose into galactose and glucose. Instead of a galactosidase, they contained a gene encoding a glucosidase, annotated as *bglC*. However, the two enzymes LacG and BglC are closely related, and in *Lactococcus lactis*, it has been shown that a glucosidase enzyme can act as a galactosidase under certain conditions [57, 58]. The dairy strain B4114 contained an additional gene within this putative *lac* operon, which is homologous (85% amino acid identity) to the *B. smithii gatA* gene, which is predicted to encode subunit IIA of a sugar phosphotransferase system [59]. The putative *lac* operon was also unique to the *G. stearothermophilus* taxon. The other dairy strains examined (*G. kaustophilus* NBRC 102445, *G. thermoglucosidasisus* strains TNO and GT23, and *G. caldoxylosilyticus* B4119) did not contain this putative *lac* operon (data not shown).

## Discussion

Traditionally the taxonomic classification of bacterial species has relied on 16S rDNA sequence analysis, DDH similarity values and phenotypic characteristics. It is challenging to classify strains to a species within the *Geobacillus* genus based solely on the 16S rRNA gene due to its high sequence similarity across the genus. It is likely that this has resulted in the mis-identification of many *Geobacillus* strains as demonstrated here and elsewhere [3, 20, 21]. Several strains analyzed in this study were previously mis-identified as *G. stearothermophilus*, for example, strain BGSC 9A21. This strain was isolated prior to the 1980s when it was believed that *G. stearothermophilus* was the only obligate thermophile of the *Bacillus* genus [60]. Although, the 16S rRNA gene sequence of this strain is approximately 98% to the type strain of *G. stearothermophilus* ATCC 12980, it is also 98–99% similar to other type strains of the *Geobacillus* genus and based on other genomic evidence is actually more closely related to *G. thermoleovorans* as demonstrated in this study. Generally isolates with <97% identity for the 16S rRNA gene are regarded as separate species [30, 33]. More recently, it was proposed that this threshold for demarcating species should be increased to 98.65% [48]. In reality, setting a threshold based on 16S rRNA gene similarity, let alone such a specific number, does not work.

The taxonomic classification of *Geobacillus* species is also uncertain, due to differences in DNA-DNA hybridization (DDH) similarity values between studies. Novel bacterial taxon descriptions also rely on phenotypic descriptions, but phenotypic characteristics may vary within a taxon. To circumvent these issues, a comparative genomics approach was taken to determine whether genome sequence data could replace the traditional methods of 16S rRNA sequence analysis, DDH, and phenotypic characteristics for defining bacterial taxa, using *G. stearothermophilus* as an exemplar.

The *Geobacillus* genus could be divided into sixteen taxa, based on both a core genome comparison and ANI, for those *Geobacillus* strains that had genome sequences available at the time of analysis. Of these, twelve appear to have validly published names *(G. caldoxylosilyticus*, *G. icigianus, G. juricassicus, G. stearothermophilus*, *G. subterraneus*, *G. thermoantarcticus*, *G. thermocatenulatus*, *G. thermodenitrificans*, *G. thermoglucosidasius*, *G. thermoleovorans, G. toebeii* and *G. vulcani*). Previous studies disagree on whether *G. thermocatenulatus* can be regarded as a separate species [21, 61, 62] and analysis of further *G. thermocatenulatus* strains as well as the type strain will be required to determine its taxonomic position. The taxonomic position of *G. zalihae* is also unclear. It bordered on the ANIm demarcation threshold from genomes in the *G. thermoleovorans* group (95.8–96.1%), although it formed a sub-group within Group 1, which may indicate that it is a subspecies of *G. thermoleovorans* rather than a separate species. In contrast, other studies describe *G. zalihae* as a genomospecies [3]. This highlights a need for clearer guidelines on how whole genome sequence analyses are interpreted to identify novel species.

In this present study, a phylogenetic network was generated for making core genome comparisons. An advantage of using a phylogenetic network, as opposed to a branching phylogenetic tree, is that it can show any ambiguous signal as to the taxonomic relationship between strains [63]. Ambiguous signal can arise from events such as gene duplication, gene transfer, different rates of mutation and recombination [64]. A comparative genomics approach used to re-examine the taxonomy of the *Geobacillus* genus demonstrated that the *Geobacillus* genus could be divided into two clades, and proposed that clade II be considered as the new genus *Parageobacillus*. This is also consistent with our results where a phylogenetic network generated using 332 core genes, showed a clear delineation between Groups 1–10 and Groups 11–16. However, distinct clades within a bacterial genus are not unusual [52, 65, 66]; separation of the *Geobacillus* genus into two genera should also be made on additional criteria, such as a discrete set of phenotypic characteristics separating the two clades. There were differences between our study and the recent analysis of Aliyu et al. [3], which compared a larger number of core genes (*n* = 1048). This is unexpected, given they examined a larger number of genomes, so the number of core genes might be expected to be lower compared with this present study. The most likely explanation is that the criteria used for defining the core genome in this present study were more stringent than that used in Aliyu et al. [3]. A core genome comparison of *Geobacillus* spp. was carried out by Studholme [30]; however, that analysis only included genome sequences in the *G. thermoleovorans, G. kaustophilus* and *G. thermocatenulatus* group. The groupings found were similar to those identified here using the OrthoMCL clustering, providing evidence that core genome comparisons are broadly comparable between research groups (although we note that Studholme [30] did not describe their method for determining the core genome).

The main focus of our study was on *G. stearothermophilus*. Compared with Groups 1–5 (Fig. 1.), *G. stearothermophilus* formed a discrete group, resulting in a clear delineation between *G. stearothermophilus* and the other *Geobacillus* taxa based on both core genome sequence analysis and ANI. Core genome sequence comparisons provided genomic evidence that the dairy strains of *G. stearothermophilus* fell within the same clade as other members of the *G. stearothermophilus* taxon. Within *G. stearothermophilus*, distinct groups were defined by both the core genome and ANI analyses, perhaps indicative of subspecies*.*

There is no one school of thought on how genomics based methodologies should be incorporated into prokaryotic taxonomy. One approach is to find a substitution for DDH, such as ANI. The use of ANI for defining new species is not without its problems [64]. Two key issues are that the genome sequences of many type strains are not available, and there are many strains that have been incorrectly identified to a given species. In the analysis of Richter and Rossello-Mora [64], it was found that for those genomes with validly published names, only 45% actually belonged to the same species as the type strain (as defined by other means such as DDH). As of 31 July 2013, there were 10,546 validly published bacterial species names, but only 14.9% of these had genome sequences available for the type strain [47]. This issue has arisen within the *Geobacillus* genus when in defining the new species *G. icigianus,* Bryanskaya et al. [19] carried out an ANI analysis, which included the genome sequences of only two type strains. In this present study, it was also shown that some genomes with validly published names did not belong to the same species as the type strain. For example, based on a *recN* sequence analysis *G. vulcani* PSS1 did not belong to the same clade as the type strain *G. vulcani* DSM 3174. Although *G. vulcani* is a validly published name, it has previously been shown to be synonymous with *G. thermoleovorans* [21] and evidence is provided here that *G. vulcani* PSS1 is a novel species, as also supported by Aliyu et al. [3].

Another issue faced when using ANI is that it takes into account the entire genome, including accessory genes. Accessory genes are generally carried by mobile elements and acquired via horizontal gene transfer as a means of adapting to a specific environment [67]. For this reason, we believe ANI is not good measure of phylogeny. Importantly, as previously expressed by others, the use of ANI in replacing DDH appears to be a case of manipulating a new method to fit an old method [49, 68], rather than taking advantage of the much greater resolution of other aspects of the new dataset.

Traditionally, a polyphasic approach, combining both genotypic and phenotypic characteristics, is used for defining new species. In incorporating a genomics approach into prokaryotic taxonomy, it has been suggested that a polyphasic approach should still be used [50]. This could not be used for *G. stearothermophilus* because of the range of phenotypic variation observed between strains. Other phenotypic characteristics such as the fatty acid content have also been shown to differ between *G. stearothermophilus* strains [54]. Importantly, discernible phenotypic characteristics are dependent on certain genes being expressed; for example, changes in the growth conditions can change the manifestation of certain phenotypic traits. Unless strict standards are in place, it can be difficult to reproduce certain phenotypic characteristics between laboratories, such as bacterial cell components (for example, fatty acids) [50].

A description of *G. stearothermophilus* has not been republished since 1986 by Claus and Berkeley [69]; therefore, Logan et al. [70] advise that this description is likely to have encompassed a variety of thermophilic bacilli strains that would now be regarded as separate taxa. In addition, it did not take into account phenotypic differences that could occur between strains as a result of adaptation of to specific environmental niches (e.g. lactose utilization).

Further evidence of this discordant use of phenotypic traits was provided by analysis of the accessory genome, where the dairy strains contained a putative *lac* operon not found in the other genomes of *G. stearothermophilus*. The presence and absence of other gene clusters required for the utilization of different carbohydrates is not unusual in the *Geobacillus* genus. Zeigler [71] analysed ten *Geobacillus* genomes and found there was variation in the number of gene clusters predicted to be involved in plant polysaccharide degradation both within and between different taxa. This supports the notions derived in this current study that inclusion of the accessory genome is not a good measure of phylogeny because of their environmental specificity and therefore should not be used for describing new species.

It has been suggested that where there are important phenotypic differences between strains of the same species (as defined by the core genome), they should be described as “biovars” of a species, instead of using phenotypic differences as a measure of taxonomy [53]. In the same study it was found that within a population of *Rhizobium leguminosarum*, the accessory genome and the ability to utilize different carbon sources differed. The authors also use the *Bacillus cereus* group, as an example, suggesting that *Bacillus anthracis* and *Bacillus thuringenisis* be named as *Bacillus cereus* biovar *anthracis* and biovar *thuringenisis* respectively. This group of bacteria show a high degree of similarity based on their chromosomal DNA, raising the question as to whether they are separate species, as they can only be differentiated by their virulence characteristics [72]. Using the biovar concept, the dairy strains of *G. stearothermophilus* could be named *G. stearothermophilus* biovar *lactis*.

## Conclusions

Two comparative genomics approaches were evaluated for their ability to define a bacterial species, in this case *G. stearothermophilus.* Both genomic approaches (core genome comparisons and ANI) grouped the twelve strains of *G. stearothermophilus* together, with the core genome comparison demonstrating variation between eleven of the strains, particularly between the dairy and non-dairy strains. Comparison of the genomes was able to resolve differences between species of the *Geobacillus* genus that cannot be determined using the traditional approach of 16S rRNA gene sequence analysis. However, although ANI was able to be used for demarcating taxa, it should not be used for determining phylogenetic relationships as it takes into account the accessory genome. When strains belonging to the same species are isolated from different environments, they may contain a different set of accessory genes as a way of adapting to a specific environment. This was seen in this present study where the dairy strains contained a unique set of genes that are probably required for lactose metabolism. A polyphasic approach for defining a bacterial species by combining genomic data with a broad range of phenotypic data would therefore not work for the *G. stearothermophilus* taxon due to the range of phenotypic variation observed between strains. Based on the findings from this study, we recommend that novel bacterial species should be defined using a core genome approach. However, for any genomic approach to become routine, all of the type strains would need to be sequenced first.

## Methods

### Genome sequences

The genome sequences of four dairy strains of *G. stearothermophilus*: three strains (A1, P3 and D1) isolated from a New Zealand milk powder manufacturing plant and one strain (B4114) isolated from buttermilk powder, [55, 56] were compared with the genome sequences of 59 other strains of *Geobacillus* (Additional file 1: Table S1) [4–7, 28, 29, 73–88]. All of the genomes were parsed and re-annotated using Prokka v. 1.10 with default parameters [89].

### Average nucleotide identity (ANI)

The ANI between two genomes has been proposed as an *in-silico* method to replace DDH [64]. This study used the default parameters in the JSpecies software package v. 1.2.1 to calculate the ANI using the program MUMmer (ANIm) between each pair of *Geobacillus* genomes. The ANIm values were used to compare the relationships between the *Geobacillus* genomes by generating a heat-map. The heat-map was generated using the heatmap.2 function included in the gplots library of the statistics software package R v. 3.2.0, visualized in Rstudio v. 0.98.1103.

### Core genome comparisons

The program OrthoMCL v. 2.0.9 [90] was used to determine the core genome. Comparison of the core genome was based on predicted amino acid sequences from ‘perfect sets’ of orthologous gene clusters (i.e., for a given gene, there were no paralogues identified within a genome), as previously described [91]. The length range of the amino acid sequences within a cluster, used in this analysis, did not vary by more than 20% of the length of the longest gene. This value allows some variation, without being too flexible, in the length of the protein amongst all cluster members. Variation in predicted protein length may occur, for example, from the actual gene starting at a different start codon from that of the predicted annotation. The core genes were aligned individually using MUSCLE v. 3.8.31 [92] and concatenated. The Neighbor-Net algorithm [93] in SplitsTree v. 4.13.1 was used to generate a Neighbor-Net with the aligned sequences.

### Phenotypic characteristics

Biochemical assays were carried out as described in Burgess et al. [54]. Motility was determined using the hanging drop method, as described by Harrigan [94], using cultures of *G. stearothermophilus* strains (A1, P3 and D1) grown in tryptic soya broth for 8 h at 55 °C.

## Additional file

Additional file 1:
**Table S1.** Lists the bacterial genomes used in this study. Table S2. This spreadsheet contains the ANIm values between each pair of genomes. (XLSX 48 kb)
